# Enhanced Arabidopsis pattern-triggered immunity by overexpression of cysteine-rich receptor-like kinases

**DOI:** 10.3389/fpls.2015.00322

**Published:** 2015-05-12

**Authors:** Yu-Hung Yeh, Yu-Hsien Chang, Pin-Yao Huang, Jing-Bo Huang, Laurent Zimmerli

**Affiliations:** Department of Life Science, Institute of Plant Biology, National Taiwan UniversityTaipei, Taiwan

**Keywords:** plant, *Arabidopsis thaliana*, cysteine-rich receptor-like kinase, innate immunity, pattern-triggered immunity, bacteria, flagellin, stomatal immunity

## Abstract

Upon recognition of microbe-associated molecular patterns (MAMPs) such as the bacterial flagellin (or the derived peptide flg22) by pattern-recognition receptors (PRRs) such as the FLAGELLIN SENSING2 (FLS2), plants activate the pattern-triggered immunity (PTI) response. The L-type lectin receptor kinase-VI.2 (LecRK-VI.2) is a positive regulator of *Arabidopsis thaliana* PTI. Cysteine-rich receptor-like kinases (CRKs) possess two copies of the C-X8-C-X2-C (DUF26) motif in their extracellular domains and are thought to be involved in plant stress resistance, but data about CRK functions are scarce. Here, we show that Arabidopsis overexpressing the LecRK-VI.2-responsive *CRK4*, *CRK6*, and *CRK36* demonstrated an enhanced PTI response and were resistant to virulent bacteria *Pseudomonas syringae* pv. *tomato* DC3000. Notably, the flg22-triggered oxidative burst was primed in *CRK4*, *CRK6*, and *CRK36* transgenics and up-regulation of the PTI-responsive gene *FLG22-INDUCED RECEPTOR-LIKE 1 (FRK1)* was potentiated upon flg22 treatment in *CRK4* and *CRK6* overexpression lines or constitutively increased by *CRK36* overexpression. PTI-mediated callose deposition was not affected by overexpression of *CRK4* and *CRK6*, while *CRK36* overexpression lines demonstrated constitutive accumulation of callose. In addition, *Pst* DC3000-mediated stomatal reopening was blocked in *CRK4* and *CRK36* overexpression lines, while overexpression of *CRK6* induced constitutive stomatal closure suggesting a strengthening of stomatal immunity. Finally, bimolecular fluorescence complementation and co-immunoprecipitation analyses in Arabidopsis protoplasts suggested that the plasma membrane localized CRK4, CRK6, and CRK36 associate with the PRR FLS2. Association with FLS2 and the observation that overexpression of *CRK4*, *CRK6*, and *CRK36* boosts specific PTI outputs and resistance to bacteria suggest a role for these CRKs in Arabidopsis innate immunity.

## Introduction

Cell surface plant receptor-like kinases (RLKs) link perception of environmental stimuli and downstream signal transductions to trigger appropriate intracellular responses. The typical structure of RLKs consists of a N-terminal receptor domain in the extracellular region, a transmembrane domain, and a C-terminal intracellular protein kinase domain (Kacperska, 2004; De Smet et al., 2009). In *Arabidopsis thaliana*, RLKs belong to a large family with more than 600 members and are classified according to their extracellular domains (Shiu and Bleecker, 2001). The best-studied RLK sub-family is characterized by the presence of leucine-rich repeat (LRR) motifs in the extracellular region. Members of this sub-family are involved in a wide range of plant signaling initiation and activation processes (Walker, 1994; Kobe and Kajava, 2001). Major examples of LRR-RLKs with known functions are BRASSINOSTEROID INSENSITIVE 1 (BRI1) that is involved in the perception of the phytohormone brassinosteroid (Wang et al., 2001; Nam and Li, 2002), CLAVATA1 that recognizes the secreted peptide CLAVATA3 to control the size of the shoot apical meristem (Clark et al., 1997; Dievart et al., 2003; Ogawa, 2008), and PEP RECEPTOR 1 (PEPR1) and PEPR2 that sense the damage-associated molecular pattern peptide 1 (Pep1) (Krol et al., 2010; Yamaguchi et al., 2010). In addition, the pattern recognition receptors (PRRs) FLAGELLIN SENSING2 (FLS2) and EF-TU receptor (EFR) are LRR-RLKs that recognize bacterial flagellin (or the derived peptide flg22) and EF-Tu (or the derived peptides elf18/elf26), respectively (Gómez-Gómez and Boller, 2000; Zipfel et al., 2006). Cell surface-localized PRRs sense conserved microbial features called microbe-associated molecular patterns (MAMPs). Recognition of MAMPs induces the pattern-triggered immunity (PTI) response characterized by the production of reactive oxygen species (ROS), activation of the mitogen-activated protein kinases (MAPKs) cascade, expression of PTI-responsive genes, callose deposition and stomatal closure (Jones and Dangl, 2006; Melotto et al., 2006; Zhang et al., 2007; Pieterse et al., 2009; Zipfel, 2009, 2014; Desclos-Theveniau et al., 2012; Kadota et al., 2014; Monaghan et al., 2014). The best-characterized PRR involved in PTI is FLS2 that recognizes the conserved microbial peptide flg22 (Gómez-Gómez and Boller, 2000). Upon flg22 elicitation, FLS2 physically associates with another LRR-RLK termed BRI1-ASSOCIATED RECEPTOR-LIKE KINASE/SOMATIC EMBRYOGENESIS RECEPTOR-LIKE KINASE3 (BAK1/SERK3), and promotes PTI responses (Chinchilla et al., 2007; Heese et al., 2007; Roux et al., 2011). Furthermore, the malectin-like LRR-RLK IMPAIRED OOMYCETE SUSCEPTIBILITY1 (IOS1) enhances PTI through the regulation of FLS2-BAK1 association (Chen et al., 2014). Another LRR-RLK, BAK1-INTERACTING RECEPTOR-LIKE KINASE 2 (BIR2) acts by negatively regulating BAK1 function to maintain immune homeostasis through the control of BAK1-FLS2 complex formation (Halter et al., 2014). Current understanding suggests that PRRs are central components of multiprotein complexes and that a dynamic regulation exists among the members of PRR complexes to fine tune the different PTI outputs (Macho and Zipfel, 2014).

In addition to LRR-RLKs, L-type lectin-RLKs (LecRKs) that are characterized by an extracellular lectin domain are known to function in plant innate immunity (Bouwmeester and Govers, 2009; Singh and Zimmerli, 2013). For example, LecRK-I.9 is a mediator of cell wall/plasma membrane adhesions and is required for resistance to pathogens via enhancement of callose deposition (Bouwmeester et al., 2011). In addition, LecRK-I.9 also known as DORN1, is necessary for eATP recognition (Choi et al., 2014). LecRK-V.5 negatively regulates stomatal immunity (Desclos-Theveniau et al., 2012) and LecRK-VI.2 is a functional protein kinase critical for Arabidopsis resistance to hemi-biotrophic *Pseudomonas syringae* pv. *tomato* DC3000 and necrotrophic *Pectobacterium carotovorum* bacteria (Singh et al., 2012a,b). Recently, the Arabidopsis LecRK-VI.2 was found to associate with FLS2 and to prime the PTI response when ectopically expressed in *Nicotiana benthamiana* (Huang et al., 2014). A genome-wide microarray analysis of an Arabidopsis *LecRK-VI.2* overexpression line revealed seven highly up-regulated cysteine-rich receptor-like kinases (CRKs) (Singh et al., 2012a). CRKs belong to a RLK sub-family with at least 44 members containing the motif with unknown function C-X8-C-X2-C (DUF26) in their extracellular domains (Chen, 2001; Wrzaczek et al., 2010). In the past decade, some CRKs were demonstrated to play critical roles in biotic and abiotic responses in Arabidopsis. For example, CRK45 also called ACRK1 was found to associate with CRK36 to negatively control ABA signaling (Tanaka et al., 2012; Zhang et al., 2013a). Besides its function in ABA-relative responses, ACRK1 also positively regulates disease resistance to *Pst* DC3000 (Zhang et al., 2013b). Similarly, CRK20 promotes conditions that are favorable for *Pst* DC3000 growth in Arabidopsis (Ederli et al., 2011). Furthermore, CRK6 and CRK7 mediate the Arabidopsis response to extracellular ROS production (Idanheimo et al., 2014), and overexpression of CRK5 increases disease resistant to *Pst* DC3000 and triggers hypersensitive response (HR)-like cell death (Chen et al., 2003). Similarly, overexpression of *CRK4*, *CRK19*, and *CRK20* that are structurally-related to CRK5 induces HR-like cell death (Chen et al., 2004), and transgenic Arabidopsis plants overexpressing *CRK13* also demonstrate enhanced resistance to *Pst* DC3000, HR-like cell death, and salicylic acid (SA) accumulation (Acharya et al., 2007). In addition to Arabidopsis CRKs, the wheat CRK *TaCRK1* is more expressed in wheat varieties resistant to *Rhizoctonia cerealis* (Yang et al., 2013). Finally, a novel antifungal protein from the endosperm of Ginkgo seeds, ginkbilobin-2 (Gnk2) that contains a conserved C-X8-C-X2-C motif, inhibits the growth of the phytopathogenic fungus *Fusarium oxysporum* (Sawano et al., 2007).

In this study, we found that overexpression of the LecRK-VI.2-responsive *CRK4*, *CRK6*, and *CRK36* increased Arabidopsis resistance to *Pst* DC3000. These CRKs were also shown to positively regulate flg22-triggered ROS production, PTI-responsive gene expression, and callose deposition, and stomatal immunity during *Pst* DC3000 infection. Exploratory experiments to identify protein-protein interaction by using co-immunoprecipitation (Co-IP) and bimolecular fluorescence complementation (BiFC) assays suggest that these CRKs are part of the PRR FLS2 protein complex. This study thus identified three CRKs positively modulating the Arabidopsis PTI response possibly through association with the PRR FLS2.

## Materials and methods

### Biological materials and growth conditions

*A. thaliana* ecotype Col-0 plants were grown in commercial potting soil/perlite (3:2) at 22–24°C day and 17–19°C night temperature under a 9-h-light/15-h-dark photoperiod. The lighting was supplied at an intensity of ~100 μE m^−2^s^−1^ by fluorescence tubes. T-DNA insertion mutants *crk4-1* (SALK_063969), *crk4-2* (SALK_089138), *crk6-1* (SALK_205955), *crk7-1* (WiscDsLox336D10), *crk7-2* (WiscDsLox502A10), *crk13-1* (SALK_085128), *crk23-1* (SALK_076541), *crk23-2* (SAIL_518_A03), *crk36-1* (SALK_035659), *crk36-2* (SALK_100834), *crk37-1* (SALK_131604), and *crk37-2* (SALK_012014) were obtained from the Arabidopsis Biological Resource Center (ABRC). Bacterial strains *Pst* DC3000 and *Pst* DC3000 *hrcC*^−^ were provided by Kunkel (Washington University, St. Louis, Missouri, USA). *Pst* bacteria were cultivated at 28°C and 220 rpm in King's B medium containing 50 mg/mL rifampicin (DC3000) or rifampicin and kanamycin (DC3000 *hrcC*^−^).

### Generation of transgenic plants

To generate transgenic Arabidopsis, the full length coding sequences (CDS) of *CRKs* were amplified by PCR with Phusion High-Fidelity DNA Polymerase (New England Biolabs) and PCR-amplified products were cloned in pCR8 TOPO TA cloning vector (Invitrogen) and TOPO-cDNA plasmids were recombined into the Gateway-compatible expression vectors pEarlyGate103 (ordered at ABRC). Pro35S: *CRKs*-*GFP* constructs were electroporated into *Agrobacterium tumefaciens* competent cells strain GV3101 and *A*. *tumefaciens* carrying Pro35S: *CRKs*-*GFP* were then used to transform Col-0 via floral drop-inoculation (Martinez-Trujillo et al., 2004). Transgenic plants were selected on MS medium containing 50 μM Glufosinate ammonium (Fluka). Multiple transgenic lines were obtained and raised to homozygotic T3 lines. All primer sequences are shown in Supplementary Table 1.

### *Pst* DC3000 bioassay

Five-week-old Arabidopsis plants were dipped in a bacterial suspension of 10^6^ colony-forming units (CFU)/mL *Pst* DC3000 in 10 mM MgSO_4_ containing 0.01% Silwet L-77 (Lehle Seeds) for 15 min. After dipping, plants were kept at 100% relative humidity overnight. Disease symptoms were evaluated at 3 days post inoculation (dpi). For bacterial titers, leaf discs collected at 2 dpi were washed twice with sterile water and homogenized in 10 mM MgSO_4_. Quantification was done by plating appropriate dilutions on King's B agar containing rifampicin (50 mg/liter) as previously described (Zimmerli et al., 2000). Each biological repeat represents nine leaf discs (0.5 cm diameter) from three different plants.

### Oxidative burst assay

Reactive Oxygen Species (ROS) evaluation was performed as previously described (Huang et al., 2012). Eight leaf discs of 0.25 cm^2^ from three 5-week-old Arabidopsis plants were incubated in 100 μL ddH_2_O overnight in 96-well plates. Water was then replaced by 100 μL of reaction solution containing 50 μM of luminol and 10 μg/mL of horseradish peroxidase (Sigma) supplemented with 50 nM of flg22 or water only for the mock controls. ROS measurements expressed as means of RLU (Relative Light Units) were conducted immediately after adding the solution with a Centro LIApcLB 692 plate luminometer (Berthold Technologies, Bad Wildbad, Germany). ROS evaluation was performed at a 2 min interval reading time for a period of 25 min.

### Stomatal movement measurement

Epidermal peels from the abaxial side of fully expanded leaves were floated in the stomatal buffer solution (25 mM MES-KOH, pH 6.15, and 10 mM KCl) under light (100 μmol m^−2^s^−1^) for at least 3 h to open stomata before stomatal aperture evaluation was performed as described (Desclos-Theveniau et al., 2012). Briefly, after treatment with 10 mM MgSO_4_ (Mock control) or bacterial suspensions (10^8^ CFU/mL *Pst* DC3000 in 10 mM MgSO_4_), pictures of stomata were taken of random regions at various time points using an Olympus BX51 microscope digital camera and application software DP2-BSW. Stomatal apertures were measured using the “measure” function of ImageJ (http://rsb.info.nih.gov/ij/). Three plants were used per biological replicates.

### RNA extraction and gene expression analysis

Total RNA was extracted and purified using the MaestroZol reagent according to the manufacturer's instructions (Omics Biotechnology Co., Ltd) with the addition of PLUS reagent for polysaccharides and proteoglycans elimination. Genomic DNA contaminations were removed using Qiagen RNase-Free DNase Set. For cDNA synthesis, 2 μg of total RNA were prepared in a volume of 22 μL DEPC-treated H_2_O and denatured at 65°C for 5 min. Eighteen point 5 mL of master mix (16 μL M-MLV buffer, 1 mM dNTP, 5 mM OligoT, 100 U M-MLV reverse transcriptase, [Invitrogen]) was added into each tube and then incubated at 37°C for 1 h, 70°C for 10 min. The cDNA was then diluted 5-times before reverse transcription-PCR (RT-PCR) or quantitative RT-PCR (qRT-PCR) analyses. RT-PCR amplification was done with 1 μL of the first-strand cDNA as template, 10 μL of Taq PLUS PCR MasterMix (#KT205, Tiangen Biotech, http://www.tiangen.com/en/) and 1 μL of 10 mM forward and reverse primers in a total volume of 20 μL. The cycling conditions were 94°C for 3 min for one initial step followed by 94°C for 30 s, 58°C for 30 s, and 72°C for 1 min, for 35 cycles. The PCR was terminated with one extra step at 72°C for 10 min. qRT-PCR were conducted on a CFX Real-Time PCR Detection System (Bio-Rad). SYBR Green fast qPCR master mix (Bio-Rad; 1 μL of cDNA, 5 μL SYBR Green supermix, 5 μL filtered sterile H_2_O, 0.5 μL of 10 mM forward and reverse primers, in a total volume of 12 μL per well) was employed for the analysis. The cycling conditions were composed of an initial 3 min denaturation step at 95°C, followed by 40 cycles at 95°C for 3 s and 60°C for 30 s. Melting curve was run from 65 to 95°C with 0.5-s time interval to ensure the specificity of product. Data were analyzed using Bio-Rad CFX manager software. *UBQ10* was used as reference gene for normalization of gene expression levels in all samples. The wild-type (WT) without any treatment or mock treatment were considered as controls in each experiments (expression level = 1). Primer sequences are shown in Supplementary Table 1.

### Callose staining

Leaves of 5-week-old Arabidopsis were syringe infiltrated with 10^8^ CFU/mL *Pst* DC3000 *hrcC*^−^ suspended in 10 mM MgSO_4_ or 500 nM flg22 dissolved in H_2_O. Control plants were respectively infiltrated with 10 mM MgSO_4_or H_2_O only. After infiltration, nine leaf discs from three different plants were selected for analyses. Harvested leaf samples were cleared overnight by incubation in 95% ethanol at room temperature and then washed three times (0.5–1 h for each washing) with sterilized H_2_O. Cleared leaves were stained with 0.01% aniline blue in 0.15 M phosphate buffer, pH 8, for 1 h. Callose deposits were visualized under UV illumination using a Nikon Optiphot-2 microscope. Callose deposition was evaluated using the “analyze particles” function of ImageJ (http://rsb.info.nih.gov/ij/).

### Subcellular localization in arabidopsis protoplast

Subcellular localization assays were performed as previously described (Chen et al., 2014). Briefly, plasmids containing 35S: *CRKs*-*GFP* or 35S: *GFP* were co-transfected with the plasma membrane marker pm-rkCD3-1007 (Nelson et al., 2007) into Arabidopsis mesophyll protoplasts by polyethylene glycol (Sigma)(Yoo et al., 2007). The samples were visualized 30 h after transfection using a TCS SP5 confocal spectral microscope imaging system (Leica).

### Bimolecular fluorescence complementation assay in arabidopsis protoplast

For BiFC assays, plasmids of Pro35S: *FLS2*-*YFP*^*N*^ (modified pEarleyGate201, Huang et al., 2014) and Pro35S: *BAK1*-*YFP*^*C*^ (modified pEarleyGate202, Huang et al., 2014), Pro35S: *FLS2*-*YFP*^*N*^ and Pro35S: *CRKs-YFP*^*C*^, Pro35S: *RCI2B-YFP*^*N*^ and Pro35S: *RCI2B-YFP*^*c*^, or Pro35S: *RCI2B-YFP*^*N*^ and Pro35S: *CRKs*-*YFP*^*C*^ were transformed into Arabidopsis protoplasts by polyethylene glycol (Sigma) for transient expression (Yoo et al., 2007). The samples were visualized after treatment with 100 nM flg22 using a TCS SP5 confocal spectral microscope imaging system (Leica).

### Co-immunoprecipitation assay in arabidopsis protoplast

For Co-IP assays, the plasmids of Pro35S: *FLS2*-*HA* (modified pEarleyGate100 with a AvrII-3xHA-SpeI fragment introduced after the attR2 recombination site) and Pro35S: *CRKs*-*GFP*, Pro35S: *FLS2*-*HA* and Pro35S: *GFP*, or Pro35S: *FLS2-HA* and Pro35S: *RBI2B-GFP* were transformed into Arabidopsis protoplasts by polyethylene glycol (Sigma) for transient expression (Yoo et al., 2007). Total proteins were extracted with 0.5 mL protein extraction buffer (50 mM Tris-HCl, pH 7.5, 150 mM NaCl, 10% glycerol, 10 mM DTT, 10 mM EDTA, 1 mM NaF, 1 mM Na_2_MoO_4_·2H_2_O, 1% [w/v] polyvinylpyrrolidone, 1% [v/v] IGEPAL CA-630 [Sigma-Aldrich] and 1% [v/v] Roche protease inhibitor cocktail) and incubated with gentle shaking at 4°C for 1 h. Samples were then centrifuged at 14000 rpm for 15 min at 4°C. Proteins were separated by SDS–PAGE and then transferred to a polyvinylidine fluoride membrane (Immobilon-P; Millipore). Supernatants (1.5 mL) were adjusted to 2 mg/mL protein and incubated for 2 h at 4°C with 20 mL GFP Trap-A beads (Chromotek). Following incubation, beads were washed four times with TBS containing 0.5% (v/v) IGEPALCA-630. Proteins were separated by 8% SDS–PAGE and then transferred to a polyvinylidine fluoride membrane (Immobilon-P; Millipore). GFP and HA fusion proteins were detected by immunoblotting with anti-GFP and anti-HA primary antibodies, respectively.

## Results

### Lines overexpressing *CRK4, CRK6*, and *CRK36* are more resistant to virulent bacteria

**Figure 1:**
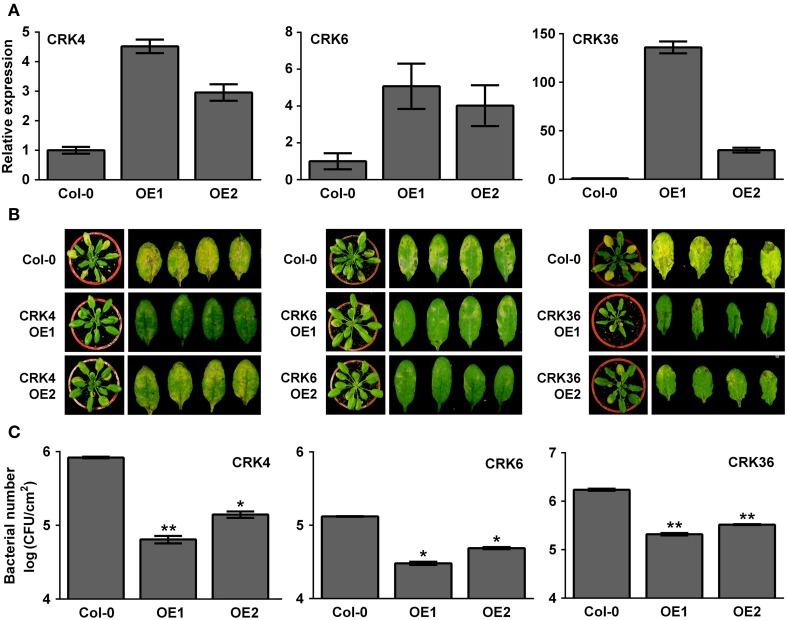
**Arabidopsis overexpressing *CRK4*, *CRK6*, and *CRK36* are more resistant to *Pst* DC3000. (A)** Expression levels of *CRK4, CRK6*, and *CRK36* in respective overexpression line 1 (OE1) and 2 (OE2). Total RNAs were extracted from 5-week-old Arabidopsis and *CRK* expression levels analyzed by qRT-PCR were expressed relatively to Col-0 WT (set at 1). *UBQ10* expression levels were used for normalization. Results are average ± SE of three biological replicates each consisting of three technical repeats (*n* = 9). **(B)** Disease symptoms in Col-0 and respective CRK overexpression line 1 (OE1) and 2 (OE2). Five-week-old plants were dip-inoculated with 10^6^ CFU/mL *Pst* DC3000 and pictures were taken 3 days later. **(C)** Bacterial growth. *Pst* DC3000 titers were evaluated at 2 dpi. Bacteria inoculation as in **(B)**. Results are average ± SE of three biological replicates each consisting of nine leaf discs (*n* = 27). Asterisks represent a significant difference to Col-0 WT (*t*-test, ^*^p < 0.05, ^**^p < 0.01).

Overexpression of *LecRK-VI.2*, a positive regulator of PTI, induces a strong up-regulation (> 10 times) of *CRK37* (At4g04500), *CRK23* (At4g23310), *CRK7* (At4g23150), *CRK6* (At4g23140), *CRK36* (At4g04490), *CRK13* (At4g23210), and *CRK4* (At3g45860) (Supplementary Table 2) (Singh et al., 2012a). To test the possible role of these CRKs in the Arabidopsis resistance response to bacterial pathogens, T-DNA insertion lines were ordered at ABRC and homozygotic mutants were tested for their resistance to the virulent bacterial pathogen *Pst* DC3000. As shown in Supplementary Figure 1, none of the tested T-DNA mutants demonstrated an altered resistance phenotype suggesting that either these CRKs are not involved in the Arabidopsis resistance to *Pst* DC3000 or functional redundancy hides CRK's role in defense against bacteria. Since most CRKs are localized in a cluster at chromosome 4, it was difficult to generate multiple CRK T-DNA insertion mutants. We thus tried to generate *CRK37*, *CRK23*, *CRK7*, *CRK6*, *CRK36*, *CRK13*, and *CRK4* overexpression lines to evaluate their resistance responses to *Pst* DC3000. Stable Arabidopsis plants overexpressing *CRK7* could not be obtained, possibly because of lethality as already observed for CRK13 (Acharya et al., 2007). Lines overexpressing *CRK13*, *CRK23*, and *CRK37* demonstrated a WT disease phenotype at 3 dpi with *Pst* DC3000 (Supplementary Figure 2). By contrast, Arabidopsis overexpressing *CRK4*, *CRK6*, and *CRK36* were more resistant to *Pst* DC3000 as illustrated by reduced disease symptoms and bacterial titers (Figure 1). These observations suggest that the *LecRK-VI.2*-responsive CRK4, CRK6, and CRK36 are involved in Arabidopsis resistance to *Pst* DC3000.

### Overexpression of *CRK4, CRK6*, and *CRK36* alters early and late PTI responses

**Figure 2:**
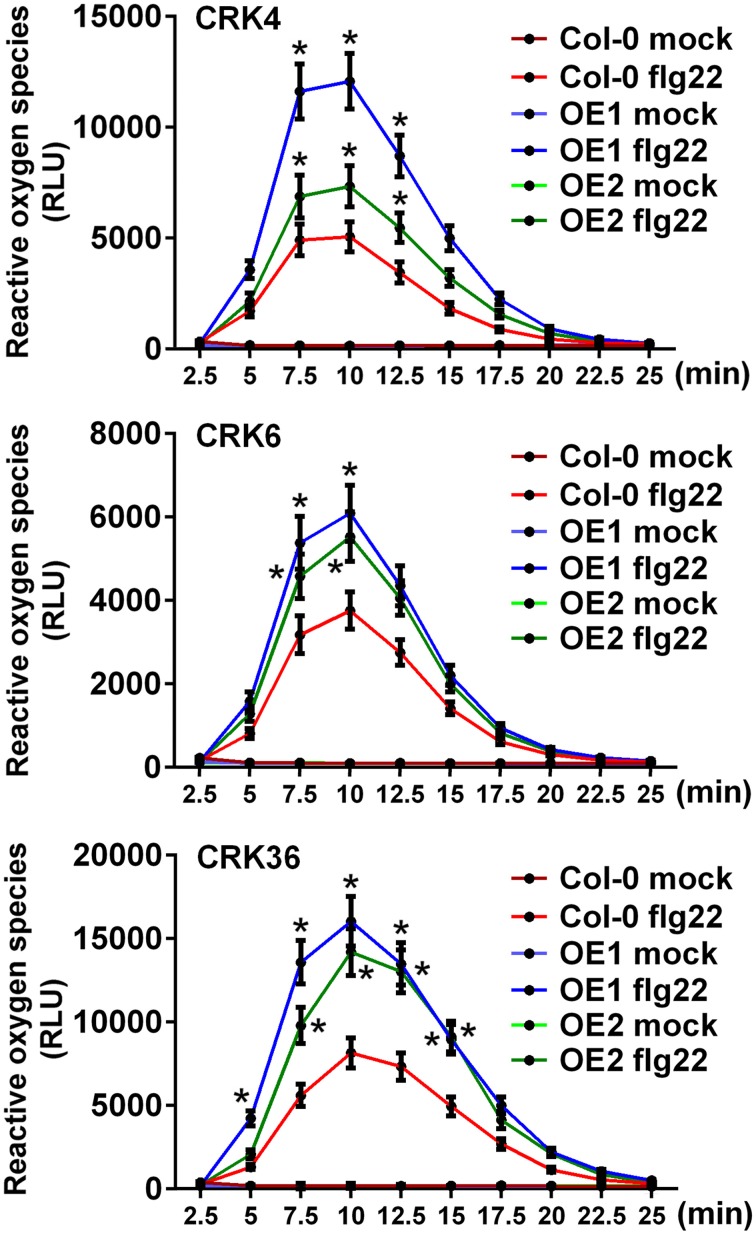
**Priming of ROS accumulation upon flg22 treatment in lines overexpressing *CRK4*, *CRK6*, and *CRK36***. ROS production represented as Relative Light Units (RLU) was evaluated for 25 min from leaf discs of three 5-week-old Arabidopsis in response to 50 nM flg22. Results represent average values ± SE of three independent biological replicates each consisting of nine leaf discs (*n* = 27). Asterisks indicate a significant difference to respective Col-0 controls (mock or flg22-treated) (*t*-test, ^*^p < 0.05).

Since LecRK-VI.2 positively regulates PTI (Singh et al., 2012a), and overexpression of the *LecRK-VI.2*-responsive *CRK4*, *CRK6*, and *CRK36* increased Arabidopsis resistance to *Pst* DC3000 (Figure 1), we hypothesized that these CRKs may positively regulate the Arabidopsis PTI response. The production of reactive oxygen species (ROS), an early PTI response (Kadota et al., 2014), was thus evaluated in lines overexpressing *CRK4*, *CRK6*, and *CRK36*. ROS production was increased in lines overexpressing these three *CRKs* after treatment with the MAMP flg22, but no constitutive ROS production was observed in mock-treated plants (Figure 2). This observation suggests priming of ROS accumulation upon PTI elicitation in *CRK4*, *CRK6*, and *CRK36* overexpression lines.

**Figure 3:**
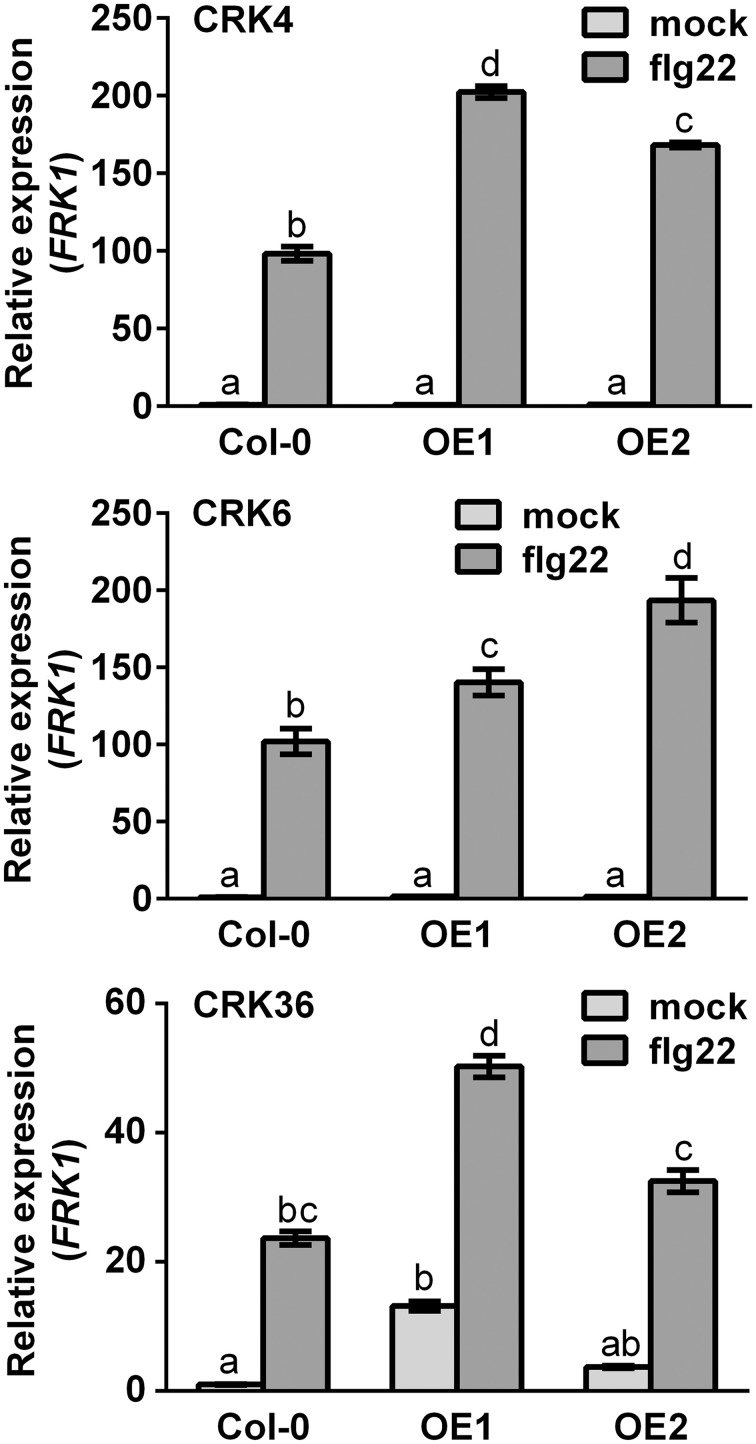
***FRK1* gene expression in *CRK4*, *CRK6*, and *CRK36* overexpression lines**. Total RNA from 10-day-old Arabidopsis seedlings was extracted at 1 h (*CRK36*) or 3 h (*CRK4* and *CRK6*) after treatment with 10 nM flg22. Relative gene expression levels were compared to mock-treated Col-0 WT (defined value of 1) by qRT-PCR analyses. *UBQ10* was used for normalization. Results represent average values ± SE of three independent biological replicates each consisting of three plants (*n* = 9). Different letters represent a significant difference (ANOVA, *p* < 0.01).

Expression levels of the PTI-responsive *FLG22-INDUCED RECEPTOR-LIKE 1* (*FRK1*), an intermediate PTI response (Asai et al., 2002), were also evaluated in CRK overexpression lines after flg22 treatment. Both *CRK4* and *CRK6* overexpression lines demonstrated a WT level of *FRK1* expression before PTI elicitation and at 1 h after flg22 treatment (Supplementary Figure 3), while *FRK1* up-regulation was significantly increased 3 h after flg22 treatment indicating potentiation of *FRK1* expression upon PTI elicitation (Figure 3). By contrast, overexpression of *CRK36* did increase *FRK1* mRNA accumulation before and at 1 h after elicitation with flg22 (Figure 3), suggesting a direct effect of this CRK on *FRK1* expression.

**Figure 4:**
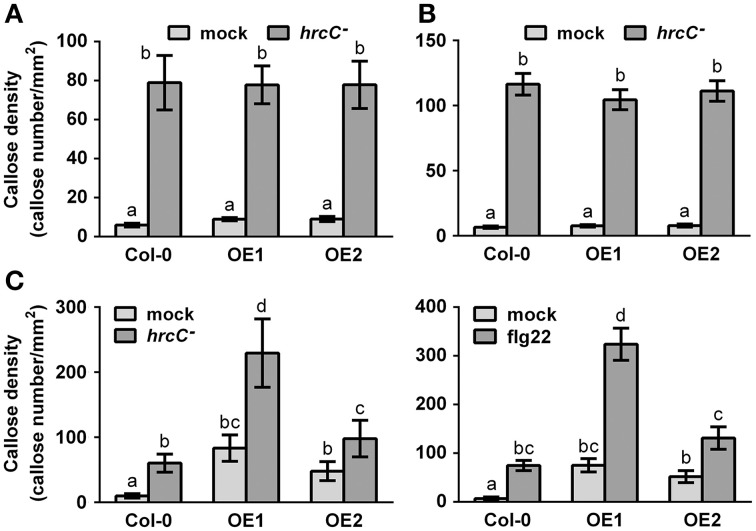
**Callose accumulation in *CRK4*, *CRK6*, and *CRK36* overexpression lines**. Callose deposits per mm^2^ in *CRK4* **(A)**, *CRK6* **(B)** and *CRK36* **(C)** overexpression lines were determined in leaf discs of three 5-week-old plants 9 h after infiltration with 10^8^ CFU/mL *Pst* DC3000 *hrcC*^−^ **(A–C)** or 500 nM flg22 **(C)**. Callose deposits were revealed by aniline blue staining. Quantification of callose accumulation was performed using the image J software. Results represent average values ± SE of three independent biological replicates each consisting of 12 leaf discs (*n* = 36). Different letters represent a significant difference (ANOVA, *p* < 0.01).

Callose deposition, a typical late PTI response (Gómez-Gómez et al., 1999), was also monitored at 9 hpi with the strain *Pst* DC3000 *hrcC*^−^, a bacterial mutant that cannot repress the PTI response because being defective in delivering type-III effectors (Brooks et al., 2004). Overexpression of *CRK4* or *CRK6* did not affect PTI-mediated callose deposition (Figures 4A,B). However, bacteria-inoculated, water- and flg22-treated *CRK36* overexpression lines accumulated more callose deposits then WT controls (Figure 4C). These observations suggest that CRK36 positively regulates callose deposition while CRK4 and CRK6 do not play a critical role in the modulation of this PTI output.

### Enhanced stomatal immunity in lines overexpressing *CRK4, CRK6*, and *CRK36*

**Figure 5:**
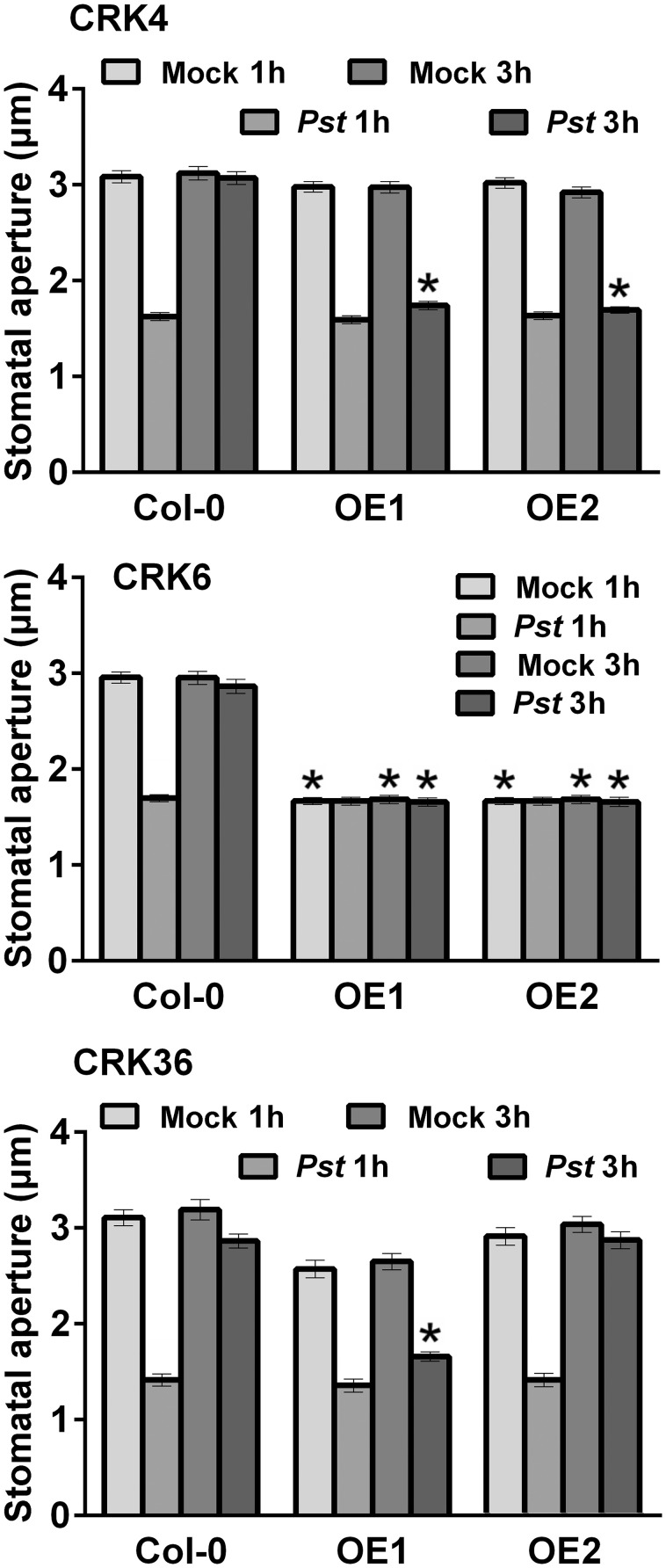
**Alteration of stomatal immunity by overexpression of *CRK4*, *CRK6*, and *CRK36***. Stomatal apertures were evaluated in leaf epidermal peels of 5-week-old Arabidopsis exposed to MgSO_4_ buffer (Mock) or *Pst* DC3000 (10^8^ CFU/mL) for 1 and 3 h. Results represent average values ± SE of three independent biological replicates each consisting of 100 technical repeats (*n* = 300). Asterisks represent a significant difference to respective Col-0 WT controls (*t*-test, ^*^p < 0.001).

As part of the PTI response, Arabidopsis plants close stomata that are in contact with bacteria (Melotto et al., 2006; Zeng et al., 2010; Desclos-Theveniau et al., 2012; Singh et al., 2012a). To enter leaves, virulent bacteria such as *Pst* DC3000 reopen stomata in a coronatine (COR)-dependent manner (Melotto et al., 2006; Zeng et al., 2010). To clarify the possible role of CRK4, CRK6, and CRK36 in stomatal immunity, stomatal apertures of CRK transgenics were evaluated at 1 and 3 hpi with *Pst* DC3000. Lines overexpressing *CRK4* or *CRK36* demonstrated a defective *Pst* DC3000-mediated stomatal reopening at 3 hpi (Figure 5). Over-expression of *CRK6* induced stomatal closure in the mock controls and at 3 hpi with *Pst* DC3000 (Figure 5). These data suggest that CRK4 and CRK36 counteract the COR-dependent reopening of stomata while CRK6 acts as a strong positive regulator of stomatal closure.

### Plasma membrane localization of CRK4, CRK6, and CRK36

**Figure 6:**
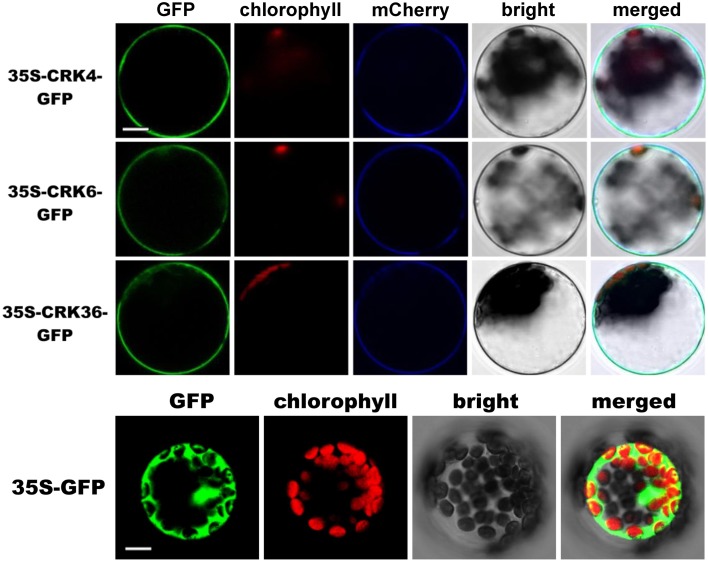
**Membrane localization of CRK4, CRK6, and CRK36**. Subcellular localization of CRK-GFP fusion proteins in Arabidopsis mesophyll protoplasts. CRK-GFP or GFP alone (negative control) constructs were transiently expressed under the cauliflower mosaic virus 35S promoter and the GFP signal was observed by confocal microscopy 16 h after transfection. The GFP fluorescence (green), the chlorophyll autofluorescence (red), the plasma membrane marker (pm-rk CD3-1007)-mCherry fluorescence localization (blue), the bright-field image, and the combined images are shown. This experiment was repeated three times with similar results. Scale bars represent 10 μm.

Subcellular localization of CRK4, CRK6, and CRK36 that possess a transmembrane domain (Chen, 2001), was performed by transiently expressing CRKs-GFP fusion protein driven by the cauliflower mosaic virus 35S promoter in Arabidopsis mesophyll protoplasts. The fluorescence signal was mainly localized at the cell periphery with a pattern similar to the plasma membrane marker pm-rk CD3-1007 (Nelson et al., 2007) (Figure 6). By contrast, the control protoplasts expressing GFP alone showed a nuclear/cytoplasmic localization (Figure 6). These observations suggest that like ACRK1 (CRK45) (Tanaka et al., 2012), CRK4, CRK6, and CRK36 are localized at the plasma membrane.

### CRK4, CRK6, and CRK36 associate with the PRR FLS2

**Figure 7:**
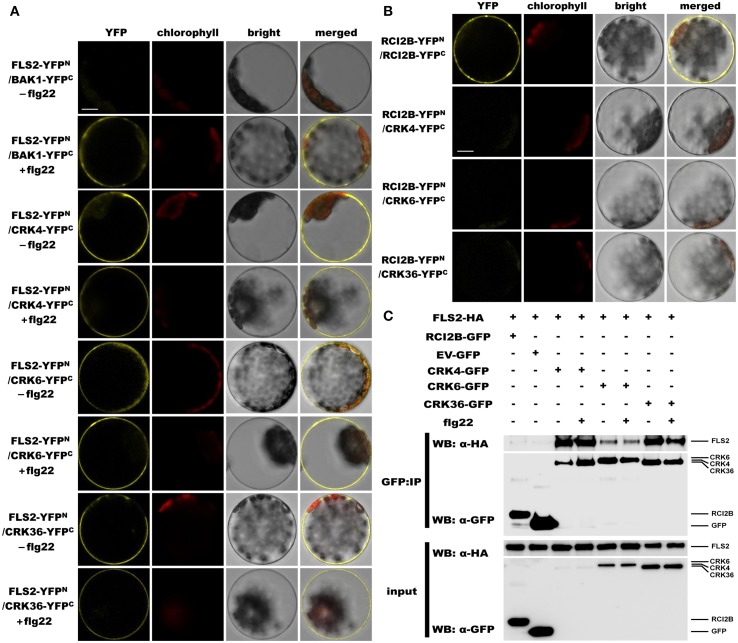
**CRK4, CRK6, and CRK36 associate with the PRR FLS2 in a flg22-independent manner. (A)** Analysis of associations between FLS2 and CRKs by BiFC assay. Arabidopsis protoplasts were co-transfected with FLS2-YFP^N^ + BAK1-YFP^C^ (positive control) or FLS2-YFP^N^ + CRKs-YFP^C^ plasmids and treated with (+) or without (−) 100 nM flg22 for 20 min. The YFP fluorescence (yellow), chlorophyll autofluorescence (red), bright field and the combined images were visualized under a confocal microscope 16 h after transfection. The scale bar represents 10 μm. **(B)** Association between RCI2B and CRKs by BiFC assay. Arabidopsis protoplasts were co-transfected with RCI2B-YFP^N^ + RCI2B-YFP^C^ (positive control), or RCI2B-YFP^N^ + CRKs-YFP^C^ plasmids and were observed by confocal microscopy 16 h after transfection. The scale bar represents 10 μm. **(C)** Analysis of association between FLS2 and CRKs by Co-IP assay. Arabidopsis protoplasts co-expressing RCI2B-GFP + FLS2-HA_3_, pG103 empty vector (EV-GFP) + FLS2-HA_3_, or CRKs-GFP + FLS2-HA_3_ constructs were treated with (+) or without (−) 100 nM flg22 for 10 min. Total protein extracts (input) and IP-proteins were detected using immunoblotting with an α-GFP or α-HA antibody. Experiments in **(A–C)** were repeated three times with similar results.

Since overexpression of *CRK4*, *CRK6*, and *CRK36* affects the early Arabidopsis PTI response, notably flg22-mediated ROS accumulation (Figure 2), we asked whether these CRKs belong to the PRR complex that recognizes the MAMP flg22. Toward this goal, we monitored CRKs association with FLS2, the PRR that recognizes flg22 (Gómez-Gómez and Boller, 2000; Chinchilla et al., 2007). Interactions were first evaluated by bimolecular fluorescence complementation (BiFC) assays (Walter et al., 2004) in Arabidopsis protoplasts. To demonstrate that our experimental conditions were appropriate, we analyzed the ligand-dependent interaction between BAK1 and FLS2 (Chinchilla et al., 2007; Heese et al., 2007; Roux et al., 2011). As expected, YFP signal was only observed after flg22 treatment (Figure 7A). When testing CRK4, CRK6, and CRK36 interactions with FLS2, the YFP fluorescence was observed in a ligand-independent manner (Figure 7A). The plasma membrane localized RARE-COLD-INDUCIBLE 2B (RCI2B)(Medina et al., 2007) also known as LTI6B (Cutler et al., 2000) was used to evaluate the specificity of CRK4, CRK6, and CRK36 association with FLS2. Importantly, both constructs were functional, as demonstrated by the clear YFP fluorescence observed in Arabidopsis protoplasts transfected with RCI2B-YFP^N^ and RCI2B-YFP^C^ (Figure 7B). However, no YFP fluorescence was observed when testing each CRK interactions with RCI2B suggesting that CRKs association with FLS2 observed by BiFC is specific (Figure 7B). Taken together, BiFC data suggest that CRK4, CRK6, and CRK36 associate with the PRR FLS2 independently of flg22 elicitation.

The Co-IP approach in Arabidopsis protoplasts was further used to evaluate the *in vivo* association of CRK4, CRK6, and CRK36 with FLS2. Toward this goal, equal amounts of CRK4, CRK6, and CRK36 were pulled down with GFP-Trap beads from Arabidopsis protoplasts transiently co-expressing CRKs-GFP and HA_3_ epitope-tagged FLS2 and detection of FLS2-HA_3_ through anti-HA immunoblotting revealed FLS2 before and after flg22 treatment (Figure 7C). It is relevant to note that the FLS2 signal after CRK6 immunoprecipitation was weaker than after CRK4 and CRK36 immunoprecipitation (Figure 7C). As a negative control, we immunoprecipitated the plasma membrane localized RCI2B protein (Medina et al., 2007) with GFP-trap beads and analyzed the presence of FLS2-HA_3_ by anti-HA immunoblotting. FLS2 could not be detected (Figure 7C). Furthermore, no signals were detected when FLS2-HA_3_ was expressed alone (Figure 7C), confirming that FSL2 does not bind aspecifically to anti-GFP magnetic beads. Together, these data suggest that CRK4, CRK6, and CRK36 associate with FLS2 in a ligand-independent manner in Arabidopsis protoplast.

## Discussion

Plant PRRs are cell-surface RLKs that trigger the PTI response through recognition of MAMPs to restrict invasion of potentially dangerous microbes (Jones and Dangl, 2006; Pieterse et al., 2009; Zipfel, 2009, 2014). Here, we characterized the function of Arabidopsis CRKs in plant innate immunity by gain-of-function analyses. The CRKs form a RLK subfamily of 44 members that are transcriptionally regulated by pathogens, ROS and SA (Chen et al., 2003, 2004; Acharya et al., 2007; Wrzaczek et al., 2010; Ederli et al., 2011). A recent genome-wide microarray analysis revealed seven CRK genes (*CRK4*, *CRK6*, *CRK7*, *CRK13*, *CRK23*, *CRK36*, and *CRK37*) that are highly up-regulated in an Arabidopsis line overexpressing the positive regulator of PTI, *LecRK-VI.2* (Singh et al., 2012a). To evaluate the role of these *CRKs* in plant defense, we screened T-DNA insertion mutants for possible altered resistance to the virulent bacterial pathogen *Pst* DC3000. None of the T-DNA mutants analyzed demonstrated a disease phenotype after *Pst* DC3000 inoculation. Since CRKs are known to compose a relatively large family, it is likely that CRKs have overlapping functions among individual members (Chen et al., 2003, 2004; Acharya et al., 2007). Therefore, no disease phenotypes in CRK T-DNA insertion mutants may be explained by functional redundancy. Transgenic plants overexpressing *CRK4*, *CRK6*, *CRK13*, *CRK23*, *CRK36*, and *CRK37* were generated to further evaluate whether these CRKs are involved in Arabidopsis disease resistance to bacteria. Arabidopsis overexpressing *CRK4*, *CRK6*, and *CRK36* demonstrated an enhanced disease resistance to *Pst* DC3000. By contrast, *CRK13*, *CRK23*, and *CRK37* overexpression did not alter Arabidopsis resistance to *Pst* DC3000. CRK13, CRK23, and CRK37 may not be involved in Arabidopsis resistance to bacteria. Surprisingly, we obtained lines stably overexpressing *CRK13* (albeit only lines with relatively low overexpression levels) while a previous work shows that lines overexpressing *CRK13* under the 35S promoter are stunted and do not survive to maturity because of lethality (Acharya et al., 2007). High overexpression of Arabidopsis *CRK13* through a steroid-inducible *Gal4* promoter induces programmed cell death (Acharya et al., 2007). Stable overexpression of *CRK13* may thus induce cell death and affects plant development and survival when highly overexpressed or under certain environment conditions. Different overexpression levels or growth conditions such as different light intensities may therefore explain the discrepancy between our results and Acharya et al. (2007) observations. Overexpression of *CRK4, CRK5, CRK19*, and *CRK20* under the control of a steroid-inducible promoter also induces spontaneous cell death (Chen et al., 2003, 2004). By contrast, none of the CRK overexpression lines analyzed in this study demonstrated a spontaneous cell death phenotype (Figure 1; Supplementary Figure 2). Possibly, only lines with low overexpression levels of CRKs involved in the regulation of program cell death were obtained in this study, since constitutive high overexpression may be lethal. For example, we could only obtain *CRK4* and *CRK6* overexpression lines with very low overexpression levels (Figure 1), suggesting a role for these CRKs in the regulation of cell death. Confirming this assumption, CRK4 is known to regulate program cell death (Chen et al., 2003, 2004). Taken together, these results support the idea that CRK4, CRK6, and CRK36 are potential players in Arabidopsis disease resistance to *Pst* DC3000.

To further characterize the role of Arabidopsis CRK4, CRK6, and CRK36 in innate immunity, we evaluated different PTI outputs in *CRK4*, *CRK6*, and *CRK36* overexpression lines. ROS accumulation is considered as an early PTI response by acting as an anti-microbial agent and/or as a secondary messenger that triggers downstream defense responses, including stomatal closure and up-regulation of PTI marker genes (Melotto et al., 2006; Kadota et al., 2014). Overexpression of *CRK4*, *CRK6*, and *CRK36* primed ROS production in response to flg22 treatment suggesting a role for these CRKs early during the activation of the PTI response. In addition, both up-regulation of *FRK1* and stomatal innate immunity were enhanced in lines overexpressing *CRK4*, *CRK6*, and *CRK36*. PTI-responsive gene up-regulation and stomatal immunity are considered as moderately early PTI outputs (Melotto et al., 2006; Singh et al., 2012a), further suggesting that these CRKs play a role at or downstream of MAMP perception. Overexpression of *CRK36* induced constitutive up-regulation of the PTI marker gene *FRK1* and callose deposition, while *FRK1* expression was primed after flg22 treatment and WT callose deposition levels were observed in *CRK4* and *CRK6* overexpression lines. CRK36 may thus function differently from CRK4 and CRK6 in the PTI signaling cascade or alternatively, higher *CRK36* relative overexpression levels may explain this divergent behavior (Figure 1). These observations suggest that like LecRK-VI.2 (Singh et al., 2012a), CRK4, CRK6, and CRK36 positively regulate some outputs of the Arabidopsis PTI response.

Early findings indicate that during pathogen infection, the Arabidopsis PRR FLS2 recognizes bacterial flagellin (or the derived peptide flg22) and induces flg22-triggered immunity (Gómez-Gómez and Boller, 2000). Currently, PRRs are believed to be central components of membrane-located multiprotein complexes that are tightly regulated for timely modulation of diverse PTI outputs (Macho and Zipfel, 2014). In this study, we identified CRK4, CRK6, and CRK36 as potential membrane-localized protein that regulate flg22-mediated PTI. We thus asked whether these CRKs are part of the PRR FLS2 complex. CRK4, CRK6, and CRK36 were found to associate *in vivo* with FLS2 in a flg22-independent manner when analyzed by BiFC and Co-IP. This observation combined with the fact that overexpression of *CRK4*, *CRK6*, and *CRK36* enhanced the production of flg22-triggered ROS, an early PTI response, suggest that these three CRKs act early during flg22-mediated PTI signaling, possibly at the PRR FLS2 complex. Protein activation by phosphorylation to regulate diverse signaling pathways is common in plants (Hardie, 1999; Nam and Li, 2002; Lu et al., 2010). Typically, BAK1 phosphorylates BIK1 after association with FLS2 and then BIK1 trans-phosphorylates FLS2 and BAK1 to activate PTI signaling (Lu et al., 2010). Since, CRK6 and CRK36 are active kinases (Tanaka et al., 2012; Idanheimo et al., 2014), they may participate in the phosphorylation cascade involved in the regulation of the PRR FSL2 complex. Since CRK proteins possess two copies of the C-X8-C-X2-C motif (Chen, 2001), CRKs could form inter-/intra-molecular disulfide bonds to strengthen the structural stability of the PRR FLS2 complex, as observed for the anti-fungal protein ginkbilobin-2 that maintains the structural stability of Gnk2 through its C-X8-C-X2-C motif by forming three intra-molecular disulfide bridges (Miyakawa et al., 2009). Collectively, this work suggests that CRK4, CRK6, and CRK36 overexpression enhances Arabidopsis resistance to *Pst* DC3000 bacteria through a positive role in the PTI response by association with the PRR FLS2. Further studies are necessary to investigate mechanistically CRK4, CRK6, and CRK36 functions at PRR complexes.

## Author contributions

LZ and YY designed the experiments. YY, YC, PH, and JH performed the experiments. LZ and YY analyzed the results and wrote the manuscript. All authors read and approved the final manuscript.

### Conflict of interest statement

The authors declare that the research was conducted in the absence of any commercial or financial relationships that could be construed as a potential conflict of interest.
