# Supplementary material for: Enhanced Arabidopsis pattern-triggered immunity by overexpression of cysteine-rich receptor-like kinases
Source: Front Plant Sci. 2015 May 12;6:322. doi: 10.3389/fpls.2015.00322 (PMC4429228; doi:10.3389/fpls.2015.00322)
Supplement: Supplementary file 1 [file DataSheet1.DOCX]

**Supplementary Material**

**
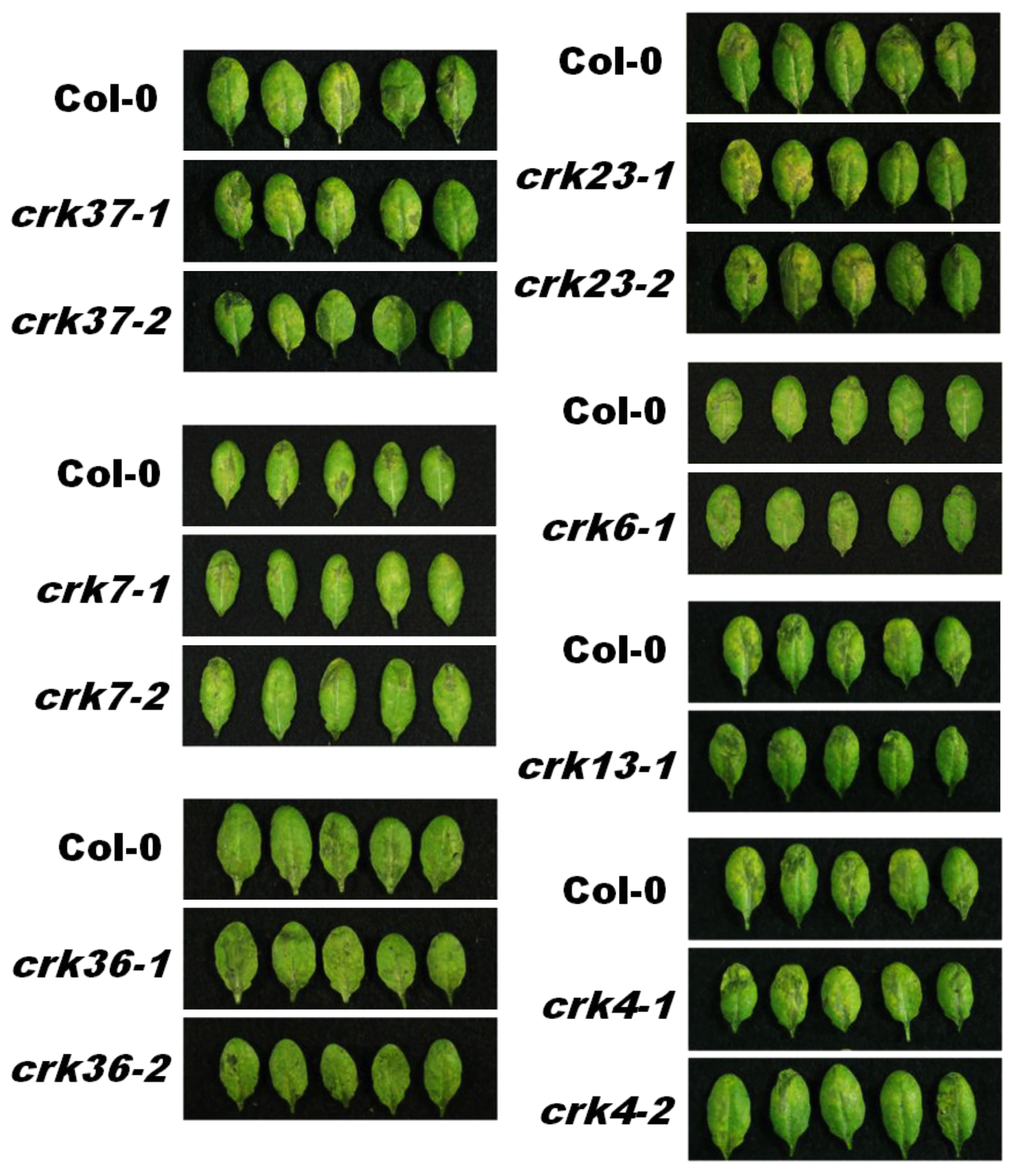
**

**SUPPLEMENTARY Figure 1. CRK37, CRK23, CRK7, CRK6, CRK13, CRK36 and CRK4 T-DNA insertion mutants demonstrate a WT resistance response to *Pst* DC3000.** Five-week-old Arabidopsis were dip-inoculated with 10^6^ CFU/mL *Pst* DC3000 and pictures of disease symptoms were taken 3 days later. All experiments were repeated 3 times with similar results.


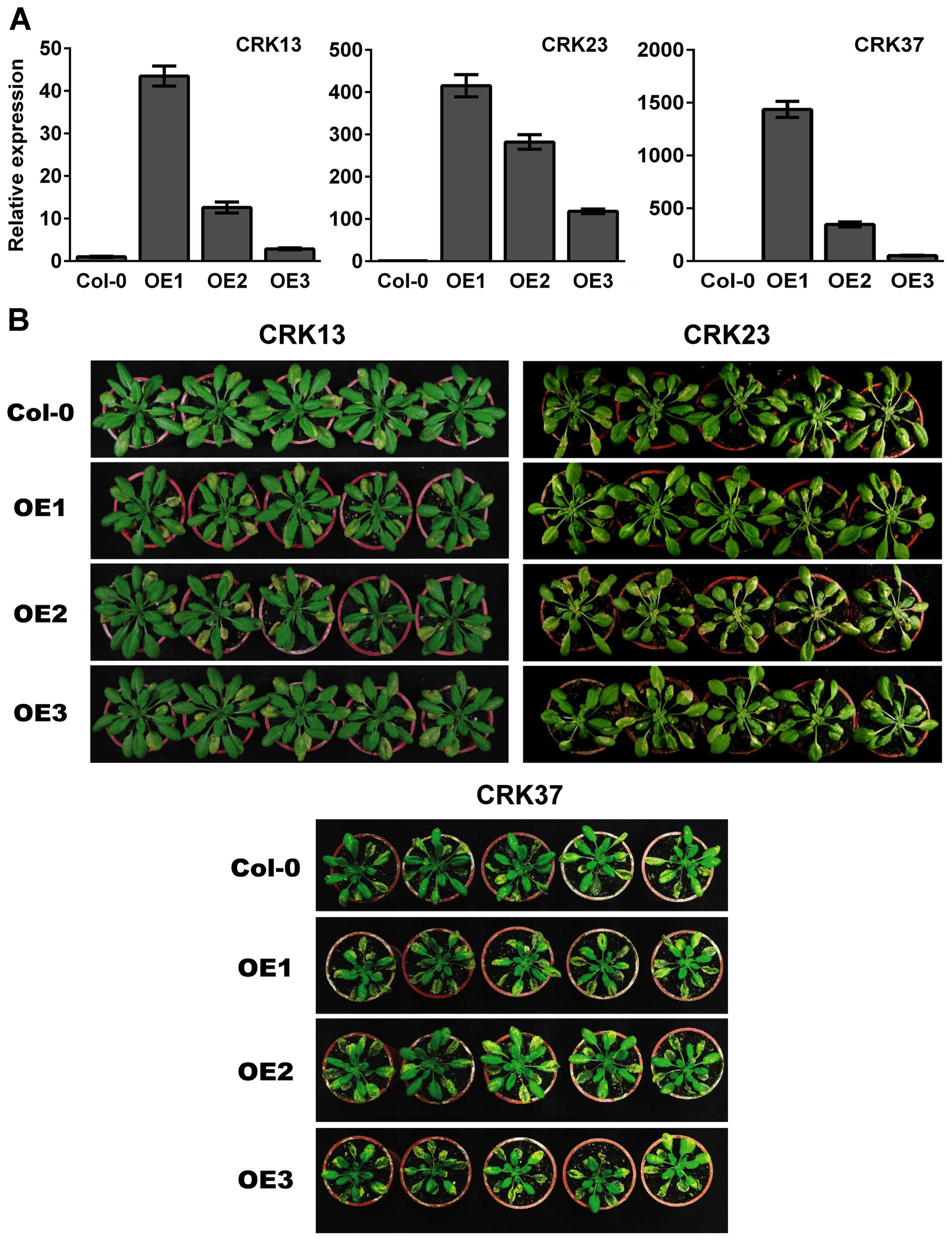


**SUPPLEMENTARY Figure 2. WT disease phenotype in *CRK13*, *CRK23*, and *CRK37* overexpression lines.** (A) *CRK13*, *CRK23* and *CRK37* expression levels in respective overexpression line 1 (OE1), line 2 (OE2) and line 3 (OE3). Total RNAs from 5-week-old Arabidopsis plants were extracted and expression levels of *CRKs* relative to Col-0 WT were analyzed by qRT-PCR. *UBQ10* was used for normalization. Results are average ± SE of 3 biological replicates each consisting of 3 technical repeats (n = 9). (B) Disease symptoms. Five-week-old plants were dip-inoculated with 10^6^ CFU/mL *Pst* DC3000 and pictures were taken 3 days later. Experiments were repeated 3 times with similar results.


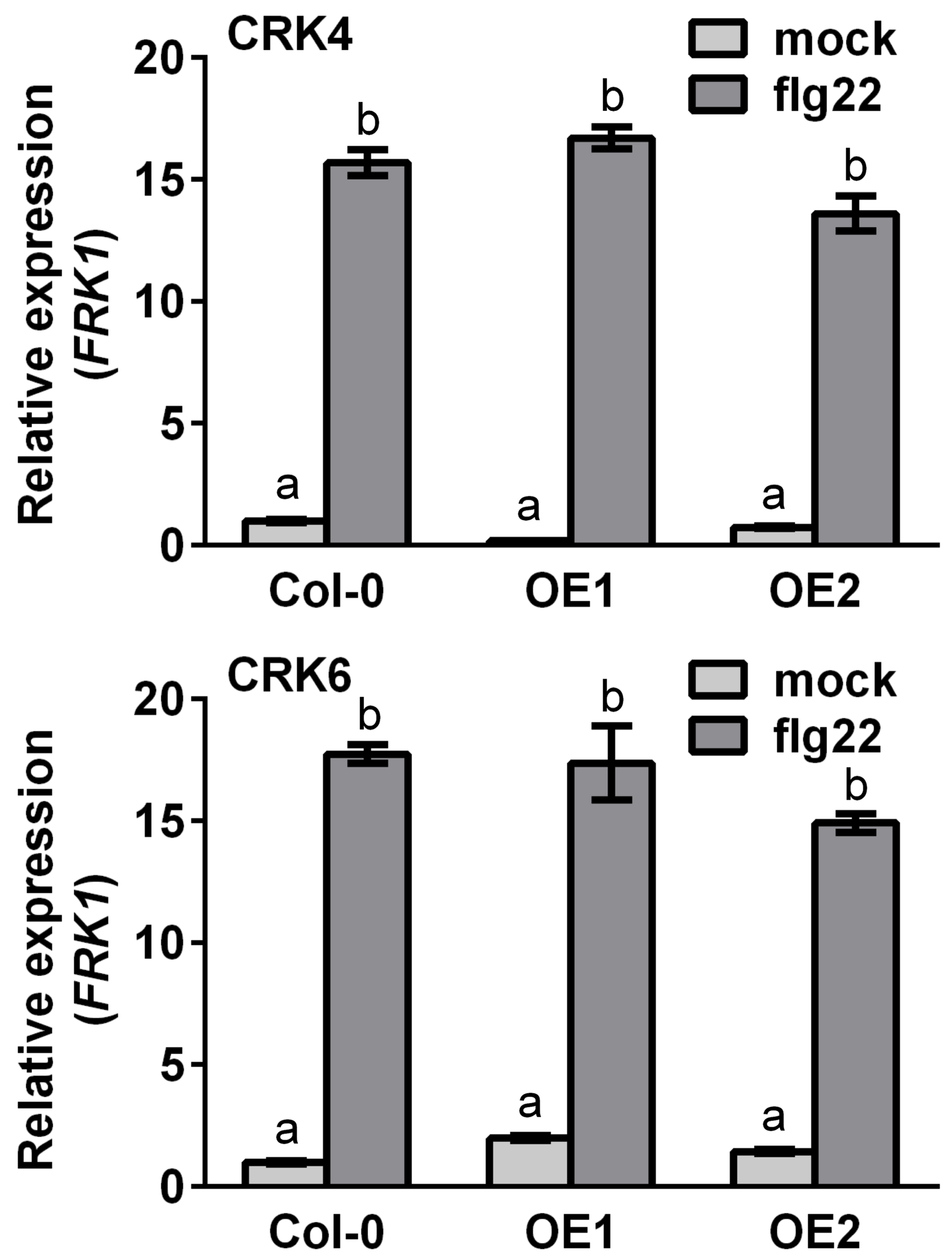


**SUPPLEMENTARY Figure 3. *FRK1* gene expression in *CRK4* and *CRK6* overexpression lines at 1 h after flg22 treatment.** Total RNA from 10-day-old Arabidopsis seedlings was extracted at 1 h after treatment with 10 nM ﬂg22. Relative gene expression levels were compared to mock-treated Col-0 WT (defined value of 1) by qRT-PCR analyses. *UBQ10* was used for normalization. Results represent average values ± SE of 3 independent biological replicates each consisting of 3 plants (n = 9). Different letters represent a significant difference (ANOVA, *p* < 0.01).

|  | **Primers used for constructs** |
| --- | --- |
| *CRK4* FP  (At3g45860) | 5'-ATGTCTTTCTTCTGGCTTTTTCC-3' |
| *CRK4* RP  (At3g45860) | 5'-ACGAGGAGTTACATTAGTAATGGAAGC-3' |
| *CRK6* FP  (At4g23140) | 5'-ATGTCTTCTC TTATATCTTT CAACTTCC-3' |
| *CRK6* RP  (At4g23140) | 5'-GCGCGGATATAAATCTGTGATCG-3' |
| *CRK7* FP  (At4g23150) | 5'-ATGTCTTCTCTCTTCCCTTTCATATTCC-3' |
| *CRK7* RP  (At4g23150) | 5'-ACGAGGATCTAAATCAGACATTG-3' |
| *CRK13* FP  (At4g23210) | 5'-ATGAAGCAGAGGAGTTTATTATCAATC-3' |
| *CRK13* RP  (At4g23210) | 5'-ATGGAACATCATTTTCAACAAGG-3' |
| *CRK23* FP  (At4g23310) | 5'-ATGTCTTCTTGGGCCTCTTTC-3' |
| *CRK23* RP  (At4g23310) | 5'-ACGAGGAGCAACACTAGTAATGGAA-3' |
| *CRK36* FP  (At4g04490) | 5'-ATGGAAAGATCCAATCTTTTCCAC-3' |
| *CRK36* RP  (At4g04490) | 5'-CCGAGGATACAAGACTGTAATCGA-3' |
| *CRK37* FP  (At4g04500) | 5'-ATGGGAAAGAGTTGTGTTGTGACTAG-3' |
| *CRK37* RP  (At4g04500) | 5'-ATGACCAAGTGATAACGATGG-3' |
|  | **Primers used for qRT-PCR** |
| *CRK4* FP  (At3g45860) | 5'-ATTGTCCAGATGCTCACTAC-3' |
| *CRK4* RP  (At3g45860) | 5'-GCGGACGTATTGATAGATAG-3' |
| *CRK6* FP  (At4g23140) | 5'-TACTGTGACCTTACCAGTGC-3' |
| *CRK6* RP  (At4g23140) | 5'-AAAAGACTTGGTCGTGGTAG-3' |
| *CRK7* FP  (At4g23150) | 5'-ACCCATATCGGTCTTTTATG-3' |
| *CRK7* RP  (At4g23150) | 5'-GTTTGTTCCAGGTCTACTCC-3' |
| *CRK13* FP  (At4g23210) | 5'-CATATGTCTGGAGGTTATGG-3' |
| *CRK13* RP  (At4g23210) | 5'-GGACGATCTGTAGGTTCTTC-3' |
| *CRK23* FP  (At4g23310) | 5'-CGCTTTACTATGTGTTCAGG-3' |
| *CRK23* RP  (At4g23310) | 5'-CTACTTGTTCATGCCTACCC-3' |
| *CRK36* FP  (At4g04490) | 5'-GGGATGTGAGGTACAGAATC-3' |
| *CRK36* RP  (At4g04490) | 5'-GCAACTTTAGGGTTCATCTC-3' |
| *CRK37* FP  (At4g04500) | 5'-TTATAGCTTTGGTGTCATGC-3' |
| *CRK37* R  (At4g04500) | 5'-ATCTCTGCAAATCTTCCTTC-3' |
| *FRK1* FP  (At2g19190) | 5'-GCCAACGGAGACATTAGAG-3' |
| *FRK1* RP (At2g19190) | 5'-CCATAACGACCTGACTCATC -3' |
| *UBQ10* FP  (At4g05320) | 5'-GGCCTTGTATAATCCCTGATGAAT -3' |
| *UBQ10* RP  (At4g05320) | 5'-AAAGAGATAACAGGAACGGAAACA-3' |

**Supplementary Table 1. Primers used in this study.** FP, forward primer. RP, reverse primer.


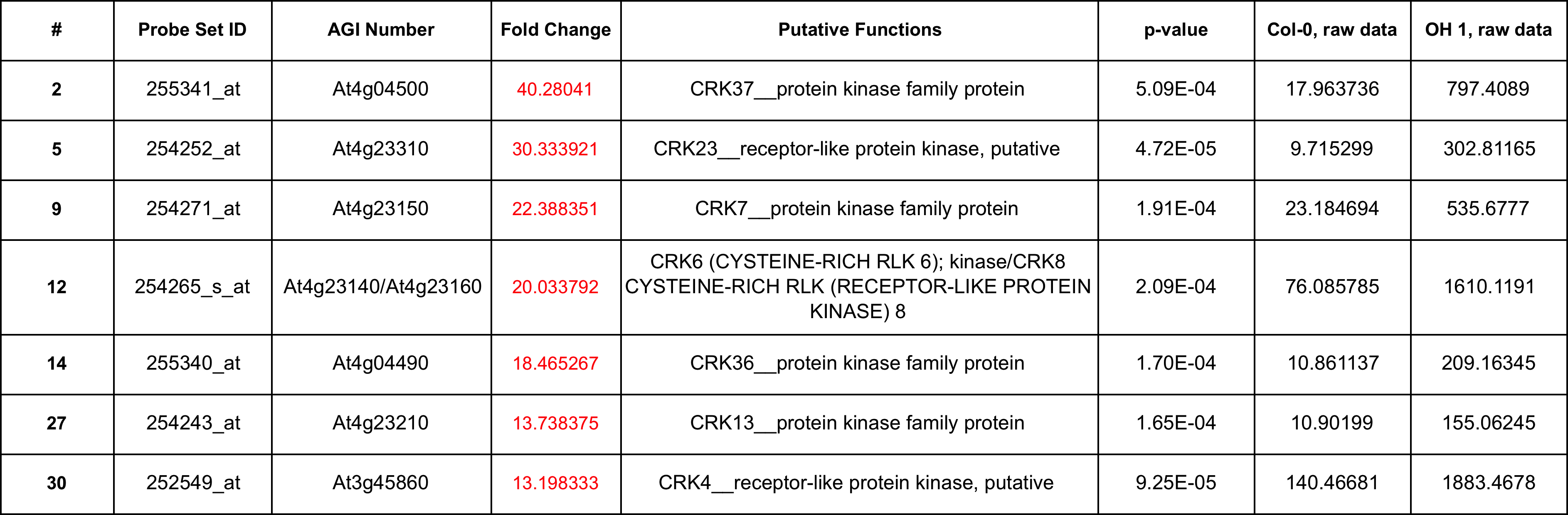


**Supplementary Table 2.** **CRKs responsive to *LecRK-VI.2* overexpression.** Up-regulated CRKs (fold change ≥ 10; P < 0.01, t-test, n=3 per group) in leaves from a line overexpressing *LecRK-VI.2* versus leaves from Col-0 control plants (from Singh et al., 2012).

**Reference**

Singh, P., Kuo, Y.C., Mishra, S., Tsai, C.H., Chien, C.C., Chen, C.W., Desclos-Theveniau, M., Chu, P.W., Schulze, B., Chinchilla, D., Boller, T., and Zimmerli, L. (2012). The Lectin Receptor Kinase-VI.2 is required for priming and positively regulates Arabidopsis pattern-triggered immunity. *Plant Cell* 24, 1256-1270.
